# Matching Biomedical Ontologies through Adaptive Multi-Modal Multi-Objective Evolutionary Algorithm

**DOI:** 10.3390/biology10121287

**Published:** 2021-12-07

**Authors:** Xingsi Xue, Pei-Wei Tsai, Yucheng Zhuang

**Affiliations:** 1 Fujian Provincial Key Laboratory of Big Data Mining and Applications, Fujian University of Technology, Fuzhou 350118, China; 2 Department of Computer Science and Software Engineering, Swinburne University of Technology, John Street, Hawthorn, VIC 3122, Australia; ptsai@swin.edu.au; 3 Intelligent Information Processing Research Center, Fujian University of Technology, Fuzhou 350118, China; 2211308002@smail.fjut.edu.cn

**Keywords:** biomedical ontology matching, multi-modal multi-objective evolutionary algorithm, guiding matrix

## Abstract

**Simple Summary:**

Biomedical ontology matching is a large-scale multi-modal multi-objective optimization problem with sparse Pareto optimal solutions. To effectively address this challenging problem, this paper proposes an adaptive multi-modal multi-Objective Evolutionary Algorithm. First, a novel multi-objective optimization model is constructed to simultaneously optimize both the alignment’s f-measure and its conservativity. Then, a problem-specific algorithm is presented, which uses the guiding matrix to adaptively guide the algorithm’s convergence and diversity in both objective and decision spaces. The experimental results show that our approach is able to effectively solve the biomedical ontology matching problem and to provide more options for decision makers.

**Abstract:**

To integrate massive amounts of heterogeneous biomedical data in biomedical ontologies and to provide more options for clinical diagnosis, this work proposes an adaptive Multi-modal Multi-Objective Evolutionary Algorithm (aMMOEA) to match two heterogeneous biomedical ontologies by finding the semantically identical concepts. In particular, we first propose two evaluation metrics on the alignment’s quality, which calculate the alignment’s statistical and its logical features, i.e., its f-measure and its conservativity. On this basis, we build a novel multi-objective optimization model for the biomedical ontology matching problem. By analyzing the essence of this problem, we point out that it is a large-scale Multi-modal Multi-objective Optimization Problem (MMOP) with sparse Pareto optimal solutions. Then, we propose a problem-specific aMMOEA to solve this problem, which uses the Guiding Matrix (GM) to adaptively guide the algorithm’s convergence and diversity in both objective and decision spaces. The experiment uses Ontology Alignment Evaluation Initiative (OAEI)’s biomedical tracks to test aMMOEA’s performance, and comparisons with two state-of-the-art MOEA-based matching techniques and OAEI’s participants show that aMMOEA is able to effectively determine diverse solutions for decision makers.

## 1. Introduction

Biomedical ontology is able to address the biomedical data heterogeneity issue and to bridge the semantic gap among multi-source and multi-modal biomedical gaps. Currently, many biomedical ontologies, such as the Systematized Nomenclature of Medicine (SNOMED) [1], the National Cancer Institute thesaurus (NCI) [2], and the Foundational Model of Anatomy (FMA) [3], have been developed to support applications such as biomedical data annotation and integration, knowledge discovery and exchange, and clinical decision support [4,5]. Biomedical research is becoming increasingly integrative in order to provide novel insights, but the need for integrating massive amounts of heterogeneous biomedical data in biomedical ontologies poses new challenge [6]. To face this challenge, it is necessary to establish the semantic correspondences for two semantic ontology-related concepts, which is the so-called biomedical ontology matching.

Due to the large-scale entities, the complex vocabularies, and the increasing semantic richness between the biomedical concepts, matching biomedical ontologies poses specific computational challenges. Recently, Evolutionary Algorithm (EA) [7] has emerged as an effective approach for optimizing the alignment’s quality. Several EAs, such as Memetic Algorithm (MA) [8] and Differential Evolution algorithm (DE) [9,10], have been used to either aggregate different ontology marchers or to directly determine all of the concept mappings. In their work, the objective was to maximize the alignment’s f-measure [11], which is a trade-off metric for recall and precision setting the aggregating weight as 0.5. However, recall and precision are two conflicting optimization objectives, and these single-objective EA-based matching techniques might improve the solutions by enhancing one of the metrics while sacrificing the other one, yielding an improvement in the solution bias. Since the matching process could be better performed by trading off different objectives instead of combining them into a single objective with the given parameters in advance, the Multi-Objective EAs (MOEAs) [12] are emerging as a popular method of optimizing the ontology alignment’s quality. MOEA is able to trade off among different objectives and to find a set of non-dominated solutions, which spread along the Pareto Front (PF) to provide more choices for DMs.

**Figure 1:**
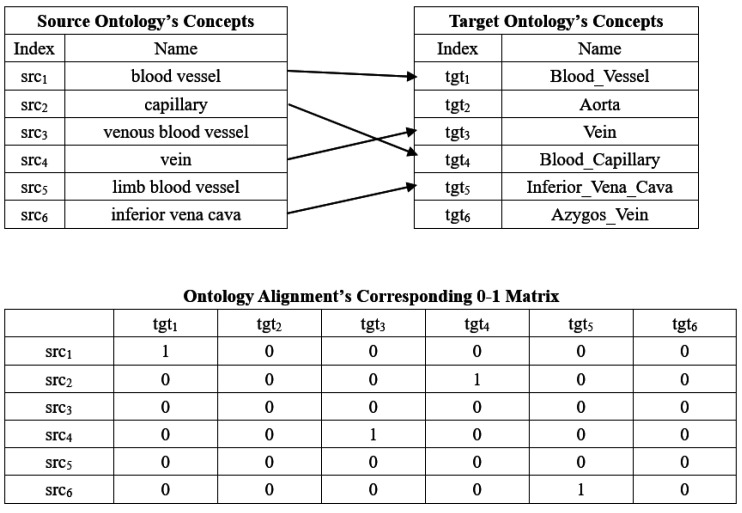
An example of the biomedical ontology alignment and the corresponding 0–1 matrix.

With respect to the biomedical ontology matching problem, there exist different solutions in the PF with the same objective values, i.e., there are several feasible regions in the decision space corresponding to the same region of an objective space, and thus, it is essentially a Multi-modal Multi-objective Optimization Problem (MMOP) [13]. It is of great significance to provide all of the Pareto sets for the Decision Makers (DMs) to provide them with more options. Currently, most MOEAs mainly focus on finding solutions with good convergence and diversity in the objective space, but for the MMOP, solutions with similar objectives of MMOP values might be diversely distributed in the decision space, which poses a challenge when solving this kind of optimization problem. In addition, solving the biomedical ontology matching problem is the processing of determining a 0–1 matrix, in which the row and column are respectively two biomedical ontologies’ concepts, and its element’s value is 1 (which means two corresponding concepts are mapped) or 0 ((which means two corresponding concepts are not regarded as a correspondence). Figure 1 shows an example of the alignment between two biomedical ontologies and the corresponding 0–1 matrix.

Since we try to find an alignment with cardinality one to one (i.e., one source concept is mapped with only one target concept and vice versa), most elements in this matrix are 0, i.e., it is a sparse matrix. Therefore, the biomedical ontology matching problem is actually a MMOP with sparse Pareto optimal solutions. To address this problem, we present a problem-specific adaptive Multi-modal MOEA algorithm (aMMOEA), which adaptively maintains several populations to execute the search process and utilizes the Guiding Matrix (GM) to adaptively guide the algorithm’s convergence and diversity in both the objective space and decision space. The main contributions made in this work are listed as follows:

- Two evaluation metrics on the alignment’s quality are proposed to calculate the alignment’s f-measure and its conservativity. On this basis, a novel multi-objective optimization model is built for the biomedical ontology matching problem;
- A problem-specific aMMOEA is presented to match two biomedical ontologies, which uses the GMs to adaptively ensure the algorithm’s convergence and diversity in both the objective space and decision space;
- The proposed aMMOEA is employed on three biomedical tracks provided by the Ontology Alignment Evaluation Initiative (OAEI) (http://oaei.ontologymatching.org, accessed on 6 December 2021); the results reveal that aMMOEA is able to effectively determine the diverse solutions for DMs.

The rest of this paper is organized as follows: Section 2 reviews EA-based ontology matching techniques; Section 3 provides preliminary background knowledge and defines the mutli-objective biomedical ontology matching problem; Section 4 presents the problem-specific aMMOEA, and Section 5 shows the experimental results; finally, conclusions and future work are given in Section 6.

## 2. Related Work

GOAL [14] is the first ontology matching system to optimize the weights of each generated similarity matrix using EA. GOAL is dedicated to addressing the meta-matching problem, i.e., how to tune the similarity measures’ aggregating weights in order to improve the alignment’s f-measure. The obtained weights could be re-used to align ontologies with the same heterogeneous characteristics. Later on, Ginsca et al. [15] optimized not only the aggregating weights but also a threshold for filtering the correspondences. Xue et al. [16] used an evaluation metric to approximately calculate the f-measure and then used a hybrid EA to integrate two ontologies’ instance sets. Acampora et al. [8] introduced a local perturbation algorithm into EA, which enhanced not only the converging speed but also the quality of solutions. For the purpose of meeting the efficiency requirements of real-time applications, Xue et al. [17] presented a Compact EA (CEA) to reduce the algorithm’s time and space complexity. Addressing the meta-matching problem should maintain the similarity matrices, which greatly increases the computational complexity. For this purpose, Wang et al. [18] modeled the matching problem as the entity matching problem, i.e., a bi-partite graph matching problem. After that, they used EA to directly determine the entity correspondences. An instance information can effectively enhance the precision of results, an instance-based similarity measure is first proposed by Alves et al. [19], and then, the hybrid EA was used to optimize the alignment. In order to further address the large-scale OM problem, Xue et al. [20,21] introduced an alignment-oriented partition algorithm that works based on the idea of divide-and-conquer. After partitioning two ontologies, they matched similar segment pairs in parallel and integrated with the greedy strategy. Chu et al. [22] first modeled two ontologies in vector space and then utilized CEA to directly determine the correspondences, which is able to improve the results’ precision value. Recently, CEA-based ontology matching techniques have also been applied to match the sensor ontologies in the Artificial Internet of Things (AIoT) [23].

The above techniques regard the ontology matching problem as the single-objective problem and then use EA to either tune the matching system’s parameters or to directly determine the alignment. Since the process of optimizing the alignment could be better performed by trading off different objectives in the matching process, recently, MOEAs have been introduced to address the ontology matching problem. Acampora et al. [24] and Xue et al. [25] both proposed to use the NSGA-II to simultaneously optimize the alignment’s recall and precision. Later on, Xue et al. [26,27] proposed to use MOEA/D to address the ontology meta-matching problem, of which the results outperforms NSGA-II based matching techniques. More recently, the meta-models were further introduced to improve the efficiency of MOEA [28,29]. Acampora et al. [30] made comparisons among the different MOEA-based matching techniques and analyzed their performance.

Different from the existing MOEA-based ontology matching techniques, this work is dedicated to addressing the multi-objective ontology entity matching problem, which is characterized as being both large-scale and multi-modal. To face this challenging problem, we use GM to guide multiple populations’ searching directions to ensure the algorithm’s convergence in objective space and diversity in both the objective space and decision space. In addition, existing approaches were dedicated to optimizing the objectives based on the alignment’s statistical features, which might lead to a logical contradiction in the final alignment. To overcome this drawback, this work optimizes both the alignment’s statistical features and its logical consistency, which is of help to further enhance the alignment’s quality.

## 3. Optimization Model on Biomedical Ontology Matching Problem

An ontology is a three tuple $\left(C,P_{d},P_{o}\right)$, where *C* is the class set, $P_{d}$ is the datatype property set, and $P_{o}$ is the object property set. Generally, class, datatype property, and object property are called ontology entities. Due to human subjectivity, ontologies in the same domain might have different ways of defining a class, yielding the ontology heterogeneity problem. Ontology matching aims to bridge the semantic gap among two ontologies by finding their entity correspondence set, i.e., the ontology alignment. In particular, an entity correspondence is a 4-tuple $\left(e_{1},e_{2},rel,conf\right)$, where $e_{1}$ and $e_{2}$ are the entities of two ontologies, rel is their relationship that could be equivalence (≡) or subsumption (⊑), and conf is the confidence degree that their relation holds. To ensure the alignment’s usefulness, the generated mapping set should reduce the logical defects according to the conservativity principle, i.e., two ontologies’ alignment should never generate new knowledge that cannot be reasoned by only one of them.

An alignment’s conservativity consists of two sub-metrics, i.e., the cardinality metric and the consistency metric [31]. With respect to the cardinality metric, since we require one-to-one alignment, i.e., one source concept is mapped with only one target concept, and vice versa, we propose MatchFmeasure [32], which is a harmony mean of MatchCoverage (a metric approximating the alignment’s recall) and the average similarity value (a metric approximating the alignment’s precision). To be specific, given an alignment *A*, its MatchFmeasure is defined as follows:

(1) $$MatchCoverage\left(A\right)=\frac{|MatchedEntity_{O_{1}}|+|MatchedEntity_{O_{2}}|}{|O_{1}\left|+\right|O_{2}|}$$

(2) $$AverageSim\left(A\right)=\frac{\sum sim_{i}}{\left|A\right|}$$

(3) $$MatchFmeasure\left(A\right)=2\times \frac{MatchCoverage(A)\times AverageSim(A)}{MatchCoverage(A)+AverageSim(A)}$$

where $|O_{1}|$, $|O_{2}|$, and *A* are, respectively, the cardinalities of ontologies $O_{1}$ and $O_{2}$, and their alignment *A*; $|MatchedEntity_{O_{1}}|$ and $|MatchedEntity_{O_{2}}|$ are the number of matched entities in two ontologies; and $sim_{i}$ is the *i*-th correspondence’s similarity value. For the similarity measure, please see our previous work [9].

Given two pairs of mappings $\left(e_{1},e_{2}\right)$ and $\left(e_{1}^{\prime },e_{2}^{\prime }\right)$, the consistency principle is described as follows: (1) if $e_{1}$ is the super-concept (or sub-concept) of $e_{1}^{\prime }$ but $e_{2}$ is not the super-concept (or sub-concept) of $e_{2}^{\prime }$, the correspondences $\left(e_{1}^{\prime },e_{2}^{\prime }\right)$ violates the consistency principle; (2) if $e_{1}$ is the super-concept (or sub-concept) of $e_{1}^{\prime }$ but $e_{2}$ is the sub-concept (or super-concept) of $e_{2}^{\prime }$, the correspondences $\left(e_{1}^{\prime },e_{2}^{\prime }\right)$ and $\left(e_{1},e_{2}\right)$ violate the consistency principle; and (3) if $e_{1}$ is the super-concept (or sub-concept) of $e_{1}^{\prime }$ and $e_{2}$ is the super-concept (or sub-concept) of $e_{2}^{\prime }$, two correspondences satisfy the consistency principle. Given an alignment *A*, its consistent subset $A^{\prime }$ in which the mappings inside satisfy the locality principle, the locality metric is defined as follows:

(4) $$consistency\left(A\right)=\frac{|A-A_{consistency}|}{\left|A\right|}$$

where |A| and $|A_{consistency}|$ are, respectively, the cardinalities of *A* and $A_{consistency}$. In this work, we first sort *A*’s correspondences in descending order according to their similarity value, then we add the mappings one by one into $A_{consistency}$ to ensure that the correspondences added later should not violate the locality principle with the ones in $A_{consistency}$.

To optimize an alignment’s quality, it is necessary to maximize an alignment Match-Fmeasure and the consistency, but they are contradictory to some extent. When we desire high consistency, we need to be selective, which will be at expense of the MatchFmeasure. Vice versa, when we want a high MatchFmeasure we have to be less selective, which will most likely decrease the consistency. To trade off these two objectives, this work models the ontology matching problem as MOP, which is defined as follows:

(5) $$\left\{\begin{array}{cc} max & f(X)=(MatchFmeasure(X),consistency(X))\\ s.t. & M_{|O_{1}\left|\times \right|O_{2}|}\\ \; & m_{ij}\in \;\left\{0,1\right\},i=1,2,\cdots ,|O_{1}\left|,j=1,2,\cdots ,\right|O_{2}| \end{array}\right.$$

where $|O_{1}|$ and $|O_{2}|$ are the cardinalities of ontologies $O_{1}$ and $O_{2}$, and $M_{|O_{1}\left|\times \right|O_{2}|}$ is an 0–1 matrix corresponding to an alignment (see also Section 1), maximizing its MatchFmeasure and consistency are two objectives.

## 4. Adaptive Multi-Modal Multi-Objective Evolutionary Algorithm

To address a large-scale MMOP with sparse Pareto optimal solutions, it is necessary to ensure the convergence of the solutions to the PF, while at the same time, maintaining the population’s diversity in both the objective and decision spaces [33]. To this end, this work presents a problem-specific aMMOEA for matching biomedical ontologies, in which the framework is presented in Algorithm 1.

In the next section, we describe the GM-based initialization, the GM-based evolutionary operators, and GM-based adaptive population maintenance.

### 4.1. Matching Matrix and Guiding Matrix

In this work, we use the MM, which is a 0–1 matrix, to encode an individual. MM’s row (or column) represents a distinct source (or target) entity, and each element represents whether the corresponding entities are mapped (with value 1) or not (with value 0). In addition, we introduce the GM, which has the same size as the MM, to describe the population’s diversity in the decision space. GM’s element is a real number in [0, 1]; when $GM_{ij}$ is close to 1, many individuals have exploited the correspondence with the *i*-th source entity and the *j*-th target entity, and when it is close to 0, the correspondences with the *i*-th source entity and the *j*-th target entity have not been explored. In each generation, we adaptively update each population’s GM and then use it to guide the algorithm’s search direction to ensure the solutions’ diversity in the decision space.
**Algorithm 1:** The framework of adaptive multi-modal multi-objective evolutionary algorithm.$SP_{num}$=2; //The number of sub-population**for** i=0; $i<SP_{num}$; *i*++ **do**    **for** j=0; $j<population_{i}.size$; *j*++ **do**        initialize(); //Initialize each sub-population    **end for****end for**Gen=0; //Current generation**while**Gen<MaximumGen**do**    **for** i=0; $i<SP_{num}$; *i*++ **do**        UpdateGM();        //GM-based evolutionary operators        crossover();        mutation();        selection();    **end for**    $SP_{num}$=MaintainPopulation(); //GM-based population maintenance    Gen=Gen+1;**end while**

### 4.2. Initialization

Given two ontologies $O_{1}$ and $O_{2}$, and a Helper Matrix (HM) $HM_{|O_{1}\left|\times \right|O_{2}|}$, an individual *x* is initialized according to Algorithm 2.

Here, we first use the similarity measure to determine the highly similar entity pairs, i.e., the anchor set. After that, teh elements in MM and HM are set as 0. According to the alignment’s consistency principle, the potential correspondence’s source entity and target entity should be the super-classes (or sub-class) of the anchor’s source entity and target entity, respectively. Therefore, we initialize the MM through reasoning with the anchor’s information. HM is used to ensure that a sub-population’s diversity in the decision space, i.e., the larger its element is, the less effort a new individual should put into the corresponding mapping in its MM.

### 4.3. Update Guiding Matrix

GM is used to guide each sub-population’s search direction, which also ensures the diversity of the whole population in the objective space. GM is updated according to the distribution of a sub-population’s solutions. Given a *i*-th sub-population, its guiding matrix $GM^{i}$ is adaptively updated as follows: first, find the non-dominated solution set NDSet in the sub-population [34]; then, build a temp matrix *M* in which the elements are set as zero; after that, for each solution x∈NDSet, find its nearest neighbor *y* in the decision space with the Hamming distance; and update each element in $M_{i}$ according to the following formula:

(6) $$M_{i,j}=\left\{\begin{array}{cc} M_{i,j}+1 & \mathrm{if}\;x_{i,j}=1\;\mathrm{and}\;y_{i,j}=1\\ M_{i,j} & \mathrm{if}\;x_{i,j}=0\;\mathrm{and}\;y_{i,j}=0\\ M_{i,j}+0.5 & \mathrm{otherwise} \end{array}\right.$$

Finally, $GM^{i}$ is updated by *M*, which is defined as follows:

(7) $$GM_{j,k}^{i}=\frac{GM_{j,k}+\frac{M_{j,k}}{\left|NDSet\right|}}{2}$$

where $GM_{j,k}^{i}$ is initialized as $\frac{M_{j,k}}{\left|NDSet\right|}$. GM ensures that the offspring solutions in one sub-population have the similar sparse distribution, and each GM is able to drive its corresponding sub-population to search for different solutions toward different directions.
**Algorithm 2:** Initialization.initialize the Anchor Set AS;initialize all the elements in *M* as zero;**if** HM is not given **then**    initialize all the elements in HM as zero;**end if****for** i=0; i<AS.length; *i*++ **do**    *m* = $AS_{i}$.sourceEntityIndex;    *n* = $AS_{i}$.targetEntityIndex;    subSet = $entity_{m}$’s sub concepts;    superSet = $entity_{m}$’s super concepts;    **for** j=0; j<rand×|subSet|; *j*++ **do**        $\left[k,k^{\prime }\right]$ = randomly select two $entity_{m}$’s sub-concept’s indices;        $\left[l,l^{\prime }\right]$ = randomly select two $entity_{n}$’s sub-concept’s indices;        **if** $HM_{k,l}<HM_{k^{\prime },l^{\prime }}$ **then**           $x_{k,l}=1$;           $HM_{k,l}=HM_{k,l}+1$;        **else**           $x_{k^{\prime },l^{\prime }}=1$;           $HM_{k^{\prime },l^{\prime }}=HM_{k^{\prime },l^{\prime }}+1$;        **end if**    **end for**    **for** j=0; j<rand×|superSet|; *j*++ **do**        $\left[k,k^{\prime }\right]$ = randomly select two $entity_{m}$’s super-concept’s indices;        $\left[l,l^{\prime }\right]$ = randomly select two $entity_{n}$’s super-concept’s indices;        **if** $HM_{K,l}<HM_{K^{\prime },l^{\prime }}$ **then**           $x_{k,l}=1$;           $HM_{k,l}=HM_{k,l}+1$;        **else**           $x_{k^{\prime },l^{\prime }}=1$;           $HM_{k^{\prime },l^{\prime }}=HM_{k^{\prime },l^{\prime }}+1$;        **end if**    **end for****end for**

### 4.4. Guiding Matrix-Based Evolutionary Operators

The GM-based crossover operator, the mutation operator, and the selection operator are respectively shown in Algorithms 3–5.

With respect to the crossover operator, the offspring individual *z* is first set as the same as with the parent solution *x*. Then, for each correspondence in the anchor set AS, in its entities’ sub-concept mappings (or super-concept mappings), when two parents are different, the corresponding element in *z* is set to 1 if a random number is smaller than GM’s corresponding elements and 0 otherwise. The crossover operator ensures that the offspring has the same genes as its two parents, and when the two parents’ gene values are different, GM is used to determine the offspring individual’s gene value. With the help of this operator, we are able to move the offspring individuals towards the PF, which ensures the sub-population’s convergence.
**Algorithm 3:** Crossover operator.[x,y]=randomly select two parents from the sub-population;z=x; //initialize the offspring individual z;**for** i=0; i<AS.length; *i*++ **do**    *m* = $AS_{i}$.sourceEntityIndex;    *n* = $AS_{i}$.targetEntityIndex;    subSet = $entity_{m}$’s sub concepts;    superSet = $entity_{m}$’s super concepts;    **for** i=0; i<subSet.length; *i*++ **do**        **for** j=0; j<GM.length; *j*++ **do**           **if** $x_{i,j}\neq y_{i,j}$ **then**               **if** $rand<GM_{i,j}$ **then**                   $z_{i,j}=1$;               **else**                   $z_{i,j}=0$;               **end if**           **end if**        **end for**    **end for**    **for** i=0; i<superSet.length; *i*++ **do**        **for** j=0; j<GM.length; *j*++ **do**           **if** $x_{i,j}\neq y_{i,j}$ **then**               **if** $rand<GM_{i,j}$ **then**                   $z_{i,j}=1$;               **else**                   $z_{i,j}=0$;               **end if**           **end if**        **end for**    **end for****end for**

The mutation operator’s two operations are executed with the same probability: for each correspondence in the anchor set AS, randomly select its entities’ sub-concepts (or super-concepts) for which the values are 1 (or 0); then, make them compete according to the GM’s corresponding elements, with the bigger (or smaller) the better; and finally, the loser’s value is flipped. In general, each offspring solution’s element is more likely to be zero if the corresponding element in the GM is smaller, and vice versa. In this way, the sub-population can generally converge towards the direction determined by the GM.

(8) $$HM_{i,j}=\left\{\begin{array}{cc} 1 & \mathrm{if}{\mathrm{HM}}_{i,j}=1\\ 1 & \mathrm{if}{\mathrm{GM}}_{i,j}>0.5\\ 0 & \mathrm{otherwise} \end{array}\right.$$

Regarding the selection operator, we used the sub-population’s unique GM to update its HM, in which the element represents whether the sub-population searched the corresponding mappings. We calculate the hamming distance between each sub-population’s individual and HM as the extra objective, which should be maximized to ensure the sub-population’s diversity in the decision space.

### 4.5. Adaptive Population Maintenance

At the end of each generation, adaptive population maintenance is executed to adjust the sub-populations. The pseudo-code of adaptive population maintenance is shown in Algorithm 6.
**Algorithm 4:** Mutation operator.**for** i=0; i<AS.length; *i*++ **do**    *m* = $AS_{i}$.sourceEntityIndex;    *n* = $AS_{i}$.targetEntityIndex;    subSet = $entity_{m}$’s sub concepts;    superSet = $entity_{m}$’s super concepts;    **if** rand<0.5 **then**        [m,n], [$m^{\prime },n^{\prime }$]=randomly select two indices from subSet, whose corresponding element’s value is 1;        **if** $GM_{m,n}<GM_{m^{\prime },n^{\prime }}$ **then**           $z_{m,n}=0$;        **else**           $z_{m^{\prime },n^{\prime }}=0$;        **end if**        [m,n], [$m^{\prime },n^{\prime }$]=randomly select two indices from superSet, whose corresponding element’s value is 1;        **if** $GM_{m,n}<GM_{m^{\prime },n^{\prime }}$ **then**           $z_{m,n}=0$;        **else**           $z_{m^{\prime },n^{\prime }}=0$;        **end if**    **else**        [m,n], [$m^{\prime },n^{\prime }$]=randomly select two indices from subSet, whose corresponding element’s value is 0;        **if** $GM_{m,n}>GM_{m^{\prime },n^{\prime }}$ **then**           $z_{m,n}=1$;        **else**           $z_{m^{\prime },n^{\prime }}=1$;        **end if**        [m,n], [$m^{\prime },n^{\prime }$]=randomly select two indices from superSet, whose corresponding element’s value is 0;        **if** $GM_{m,n}>GM_{m^{\prime },n^{\prime }}$ **then**           $z_{m,n}=1$;        **else**           $z_{m^{\prime },n^{\prime }}=1$;        **end if**    **end if****end for**

The smaller hamming distance between two sub-populations indicates a larger overlap between their search directions, and one of the sub-populations should be deleted. In contrast, if their similarity are small and all of the sub-populations have non-dominated individuals, a new sub-population will be added. Otherwise, no sub-population will be added or deleted. In particular, adaptive population maintenance aims at diversifying the search directions of sub-populations to find more equivalent non-dominated optimal solutions.
**Algorithm 5:** Selection operator.update the Helper Matrix HM according to Equation (8);**for** each individual ind in the sub-population **do**    addNewObjective(HammingDistance(ind,HM));**end for**[$F_{1},F_{2},\cdots$]=NonDominatedSort();$k=min_{i}\left|F_{1}F_{2}\right|>$*PopulationSize*;$F_{k}$ = delete();$subPopulation=F_{1}F_{2}F_{k}$;

**Algorithm 6:** Adaptive population maintenance.
[$SP_{a}$, $SP_{b}$] = Select two most similar sub-populations according to the hamming distance between their GMs;**if** $HammingDistance(GM_{a},GM_{b}$)<0.5 **then**    //delete a sub-population    $SP_{a}=selection\left(SP_{a}SP_{b},PopulationSize/\left(SP_{num}-1\right)\right)$;    delete $SP_{b}$;    $SP_{num}=SP_{num}-1$;
**else**
    **if** all sub-populations have non-dominated individuals **then**        //add a new sub-population        **for** each sub-population $SP_{i}$ **do**           $SP_{i}=selection\left(SP_{i},PopulationSize/\left(SP_{num}+1\right)\right)$;        **end for**        initialize $SP_{SP_{num}+1}$ with $HM_{K}=\sum_{i=1}^{SP_{num}}$;        $SP_{num}=SP_{num}+1$;    **end if**
**end if**



## 5. Experiment

### 5.1. Experimental Setup

The experiment tests aMMOA’s performance with OAEI’s biomedical tracks, which are regarded as the authorized testing cases for evaluating the biomedical ontology matching technique’s performance. In Table 1, the test cases are briefly described.

In Table 1, all of the test cases are from real biomedical projects, and the ontologies used are famous ones in the biomedical domain. The anatomy track consists of two biomedical ontologies that describe the adult Mouse Anatomy (MA) (http://www.informatics.jax.org/searches/AMA_form.shtml, accessed on 6 December 2021) and Human Anatomy (HA) (www.cancer.gov/cancertopics/cancerlibrary/terminologyresources, accessed on 6 December 2021). MA is provided by Mouse Genome Informatics (MGI) (http://www.informatics.jax.org/mgihome/projects/aboutmgi.shtml, accessed on 6 December 2021), which is an international database resource for the laboratory mouse, providing integrated genetic, genomic, and biological data to facilitate the study of human health and disease, and HA is maintained by National Cancer Institute Center for Biomedical Informatics and Information Technology (https://datascience.cancer.gov/, accessed on 6 December 2021), which provides the vocabularies for cancer research. The large biomed track aims to find alignments between three large and semantically rich biomedical ontologies, i.e., Systematized Nomenclature of Medicine (SNOMED) [1], the National Cancer Institute thesaurus (NCI) [2], and the Foundational Model of Anatomy (FMA) [3]. SNOMED is designed as a comprehensive nomenclature of clinical medicine for the purpose of accurately storing and/or retrieving records of clinical care in human and veterinary medicine. NCI is the oldest and largest research program of the 27 institutes and centers of the NIH, which maintains the thesaurus to support the scientific research, health information dissemination, and other activities related to the causes, prevention, diagnosis, and treatment of cancer. FMA is developed and maintained by the Structural Informatics Group at the University of Washington, which is a reference ontology for the domain of Human anatomy, which is a symbolic representation of the canonical, phenotypic structure of an organism. The disease and phenotype track has two test cases that involve four biomedical ontologies covering the disease and phenotype domains. The Human Phenotype Ontology (HP) (https://hpo.jax.org, accessed on 6 December 2021) is a standardized vocabulary of phenotypic abnormalities that have been seen in human disease, of which the data can be used for clinical diagnostics, for mapping between phenotypes of model organisms, and as a standard vocabulary for clinical database. The Human Disease Ontology (DOID) (https://disease-ontology.org/, accessed on 6 December 2021) was developed in 2003 at Northwestern University to address the need for a purpose-built ontology that covers the full spectrum of disease concepts annotated within biomedical repositories within an ontological framework that is extensible to meet community needs. Mammalian Phenotype Ontology (MP) (http://www.informatics.jax.org/vocab/mp_ontology, accessed on 6 December 2021) was developed by MGI, which describes the terminologies on the observable morphological, physiological, behavioral, and other characteristics of mammalian organisms that are manifested through development and lifespan. Orphanet and Rare Diseases Ontology (ORDO) (https://www.ebi.ac.uk/ols/ontologies/ordo, accessed on 6 December 2021) was jointly developed by Orphanet and the EBI to provide a structured vocabulary for rare diseases capturing relationships between diseases, genes, and other relevant features and forms a useful resource for the computational analysis of rare diseases.

In the experiment, we compare the NSGA-II [35] and MOEA/D [26]-based ontology matching techniques and OAEI participants. The configurations of NSGA-II and MOEA/D are given in Table 2. The specific evolutionary operators are referenced from their literature. The knee solution is such a particular solution on the PF that the improvement of any one of its objectives yields significant deterioration on the others. Since there is a link between the knee solutions in bi-criteria problems and the preferred methodologies when viewed from a conflicting bi-criterion [36], in this paper, we take three knee solutions in the PF as the output of MOEA, i.e., the solutions with the best f-measure, the best precision, and the best recall.

### 5.2. Experimental Results

Table 3 compares three MOEA-based matching technique in terms of the alignment’s quality. In particular, we present their solutions with the best f-measure, recall, and precision and the corresponding standard deviation values. Table 4 and Table 5 present the T-test statistical analysis [37] on the values in Table 3. Table 6 compares aMMOEA with state-of-the-art ontology matching systems.

Since aMMOEA maintains the diversity of solutions in both the objective space and decision space and takes recall into consideration, it is more likely to provide better solutions than NSGA-II and MOEA/D, which consider diversity in the objective space only. In addition, the NSGA-II and MOEA/D-based matching techniques aim to address the ontology meta-matching problem, which optimizes the aggregating weights for different similarity measures. When dealing with a large-scale issue, such as the biomedical ontology matching problem, it is difficult to ensure their convergence to the PF. AMMOEA introduces the GM to adaptively ensure the algorithm’s convergence, which is able to effectively determine high-quality solutions. It can be seen from Table 3, Table 4 and Table 5 that aMMOEA’s statistically outperforms the other two MOEA-based matching techniques on all test cases at the 5% significance level. Finally, the two existing MOEA-based matching techniques do not take into consideration the conservativity of the alignment, while this work maximizes the alignment’s conservativity as one of the objectives and is able to ensure the solutions’ convergence to the true PF. In Table 6, aMMOEA’s solutions with the best f-measure are also generally better than the other state-of-the-art ontology matching systems. To conclude, the aMMOEA-based biomedical ontology matching technique is able to effectively determine diverse solutions and to provide more options for the decision maker.

### 5.3. Computational Complexity on Adaptive Multi-Modal Multi-Objective Evolutionary Algorithm

The time complexity of non-dominated sorting is $O(N^{2})$, where *N* is the population size. The time complexity of updating GM and of generating the new individual are both K×O(N/K)D=O(ND), where *K* and *D* are, respectively, the number of sub-populations and decision variables. With respect to the time complexity of the selection operator and the adaptive population maintenance, Hamming distance calculation has a time complexity of O(N/K)D=O(ND), the non-dominated sorting’s complexity here is $K\times O{\left(N/K\right)}^{2})=O\left(N^{2}/K\right)$, and the crowd distance calculation’s time complexity is K×O((N/K)logN/K)=O(NlogN/K). To sum up, the total time complexity of aMMOEA is O(MaximumGen×N(N+D)).

## 6. Conclusions and Future Work

Due to the large-scale entities, the complex vocabularies, and the increasing semantic richness between the biomedical concepts, effectively determining high-quality biomedical alignment is a challenge. To face this challenge, this work proposes an aMMOEA-based biomedical ontology matching technique. In particular, we first construct a novel optimization model to define the biomedical ontology matching problem. After analyzing this problem’s essence, we present a problem-specific aMMOEA to address it. The proposed aMMOEA uses the GM to adaptively guide the algorithm’s sub-populations’ search directions, which is able to ensure the solutions’ convergence in the objective space and diversity in both the objective and decision spaces. The experiment uses OAEI’s biomedical tracks to test aMMOEA’s performance, and the experimental results show that aMMOEA is able to effectively match the biomedical ontologies and to provide diverse options for DMs.

In the future, we are interested in further improving aMMOEA by finding the complex ontology alignment, i.e., the cardinality of the alignment could be many to many and the relationships between the entities could be subsumption. Additionally, the efficient ontology partition algorithm could be used to convert the large-scale problem into small-scale ones and is able to reduce the matching process’s memory consumption and run time. To facilitate the application of biomedical ontology alignments in the clinical diagnosis, we need to develop a simple but clear way of explaining the alignment for decision makers.

## Figures and Tables

**Table 1:** Brief description on OAEI’s biomedical tracks.

Track ID	Ontologies	Tasks
Anatomy Track (http://oaei.ontologymatching.org/2021/anatomy/index.html, accessed on 6 December 2021)	Adult Mouse Anatomy (MA)-2744 classes Human Anatomy (HA)-3304 classes	MA-HA
Large Biomed Track (http://www.cs.ox.ac.uk/isg/projects/SEALS/oaei/, accessed on 6 December 2021)	Foundation Model of Anatomy (FMA)-78,989 classes Systemized Nomenclature of Medicine (SNOMED)-122,464 classes National Cancer Institute thesaurus (NCI)-66,724 classes	FMA-NCI FMA-SNOMED SNOMED-NCI
Disease and Phenotype Track (https://sws.ifi.uio.no/oaei/phenotype/, accessed on 6 December 2021)	Human Phenotype Ontology (HP)-33,205 classes Mammalian Phenotype Ontology (MP)-32,298 classes	HP-MP
	Human Disease Ontology (DOID)-24,034 classes Orphanet Rare Disease Ontology (ORDO)-68,009 classes	DOID-ORDO

**Table 2:** The parameters used by NSGA-II and MOEA/D.

	Population Size	Selection Rate	Crossover Rate	Mutation Rate	Maximum Generations
NSGA-II	201	0.8	0.98	0.05	300
MOEA/D	201	0.8	0.98	0.05	300

**Table 3:** Comparison among three MOEA-based matching techniques in terms of best f-measure (best recall and best precision) and standard deviation. The symbols *f*, *r*, *p*, and stDev stand for f-measure, recall, precision, and standard deviation, respectively.

ine Testing Case	NSGA-II		MOEA/D		aMMOEA	
ine	f (r, p)	stDev	f (p, r)	stDev	f (p, r)	stDev
ine Anatomy	0.85 (0.89, 0.76)	0.02 (0.02, 0.02)	0.85 (0.89, 0.84)	0.02 (0.02, 0.01)	0.92 (0.94, 0.96)	0.01 (0.02, 0.01)
FMA-NCI	0.87 (0.86, 0.86)	0.02 (0.02, 0.02)	0.84 (0.88, 0.78)	0.02 (0.02, 0.02)	0.93 (0.95, 0.98)	0.02 (0.02, 0.02)
FMA-SNOMED	0.71 (0.77, 0.63)	0.02 (0.02, 0.01)	0.65 (0.62, 0.75)	0.01 (0.01, 0.01)	0.84 (0.86, 0.88)	0.01 (0.02, 0.01)
NCI-SNOMED	0.68 (0.69, 0.64)	0.01 (0.01, 0.02)	0.68 (0.65, 0.70)	0.02 (0.02, 0.03)	0.77 (0.77, 0.80)	0.01 (0.01, 0.01)
HP-MP	0.55 (0.47, 0.57)	0.02 (0.02, 0.01)	0.71 (0.68, 0.72)	0.02 (0.02, 0.02)	0.85 (0.78, 0.89)	0.01 (0.01, 0.02)
DOID-ORDO	0.81 (0.83, 0.80)	0.01 (0.01, 0.01)	0.84 (0.83, 0.85)	0.02 (0.03, 0.01)	0.93 (0.93, 0.97)	0.02 (0.02, 0.02)
ine						

**Table 4:** *t*-Test’s *t*-value on the alignment’s quality.

ine Testing Case	*t*-Value	*t*-Value
ine	(NSGA-II, aMMOEA)	(MOEA/D, aMMOEA)
	f-measure (recall, precision)	f-measure (recall, precision)
ine Anatomy	−17.14 (−9.68, −48.98)	−17.14 (−9.68, −46.47)
FMA-NCI	−11.61 (−17.42, −23.23)	−17.42 (−13.55, −38.72)
FMA-SNOMED	−31.84 (−17.42, −6.82)	−73.58 (−58.78, −50.34)
NCI-SNOMED	−34.85 (−30.98, −39.19)	−22.04 (−29.39, −17.32)
HP-MP	−73.48 (−75.93, −78.38)	−34.29 (−24.49, −32.92)
DOID-ORDO	−29.39 (−24.49, −41.64)	−17.42 (−15.19, −29.39)
ine		

**Table 5:** *t*-Test’s *p*-value on the alignment’s quality.

ine Testing Case	*p*-Value	*p*-Value
ine	(NSGA-II, aMMOEA)	(MOEA/D, aMMOEA)
	f-measure (recall, precision)	f-measure (recall, precision)
ine Anatomy	0.0185 (0.0327, 0.0064)	0.0185 (0.0327, 0.0068)
FMA-NCI	0.0273 (0.0182, 0.0136)	0.0182 (0.0234, 0.0082)
FMA-SNOMED	0.0099 (0.0182, 0.0463)	0.0043 (0.0054, 0.0063)
NCI-SNOMED	0.0091 (0.0102, 0.0051)	0.0144 (0.0108, 0.0183)
HP-MP	0.0043 (0.0041, 0.0040)	0.0092 (0.0129, 0.0096)
DOID-ORDO	0.0108 (0.0129, 0.0076)	0.0182 (0.0209, 0.0108)
ine		

**Table 6:** Comparison with OAEI participants in terms of f-measure.

ine Testing Case	AML	LogMap	XMap	DOME	POMAP++	aMMOEA
ine Anatomy	0.94	0.89	0.89	0.76	0.89	0.92
FMA-NCI	0.93	0.92	0.86	0.86	0.88	0.93
FMA-SNOMED	0.83	0.79	0.77	0.33	0.40	0.84
NCI-SNOMED	0.80	0.77	0.69	0.64	0.68	0.77
HP-MP	0.84	0.85	0.47	0.47	0.68	0.85
DOID-ORDO	0.64	0.84	0.70	0.60	0.83	0.93
ine Average	0.83	0.84	0.73	0.61	0.72	0.87
ine						

## Data Availability

The data presented in this study are available from the corresponding author upon request.
